# Mobilizing health district management teams through digital tools: Lessons from the District.Team initiative in Benin and Guinea using an action research methodology

**DOI:** 10.1002/lrh2.10244

**Published:** 2020-08-28

**Authors:** Basile Keugoung, Kéfilath Olatoyossi Akankè Bello, Tamba Mina Millimouno, Sidikiba Sidibé, Jean Paul Dossou, Alexandre Delamou, Antoine Legrand, Pierre Massat, Nimer Ortuno Gutierrez, Bruno Meessen

**Affiliations:** ^1^ Health Service Delivery Community of Practice Yaounde Cameroon; ^2^ Centre de Recherche en Reproduction Humaine et en Démographie Cotonou Benin; ^3^ Centre National de Formation et de Recherche en Santé Rurale de Maferinyah Forécariah Guinea; ^4^ Blue Square Brussels Belgium; ^5^ Mavromatika Antwerp Belgium; ^6^ Health Research Unit Action Damien Brussels Belgium; ^7^ Collective Horizon Lier Belgium; ^8^ Public Health Department Institute of Tropical Medicine Antwerp Belgium

**Keywords:** action research, Benin, community of practice, Guinea, health district, mobilization

## Abstract

**Background:**

Improving capacities of health systems to quickly respond to emerging health issues, requires a health information system (HIS) that facilitates evidence‐informed decision‐making at the operational level. In many sub‐Saharan African countries, HIS are mostly designed to feed decision‐making purposes at the central level with limited feedback and capabilities to take action from data at the operational level. This article presents the case of an eHealth innovation designed to capacitate health district management teams (HDMTs) through participatory evidence production and peer‐to‐peer exchange.

**Methods:**

We used an action research design to develop the eHealth initiative called “District.Team,” a web‐based and facilitated platform targeting HDMTs that was tested in Benin and Guinea from January 2016 to September 2017. On District.Team, rounds of knowledge sharing processes were organized into cycles of five steps. Quantitative and qualitative data were collected to assess the participation of HDMTs and identify enablers and barriers of using District.Team.

**Results:**

Participation of HDMTs in District.Team varied between cycles and steps. In Benin, 79% to 94% of HDMTs filled in the online questionnaire per cycle compared to 61% to 100% in Guinea per cycle. In Benin, 26% to 41% of HDMTs shared a commentary on the results published on the platform while 21% to 47% participated in the online discussion forum. In Guinea, only 3% to 8% of HDMTs shared a commentary on the results published on the platform while 8% to 74% participated in the online discussion forum. Five groups of factors affected the participation: characteristics of the digital tools, the quality of the facilitation, profile of participants, shared content and data, and finally support from health authorities.

**Conclusion:**

District.Team has shown that knowledge management platforms and processes valuing horizontal knowledge sharing among peers at the decentralized level of health systems are feasible in limited resource settings.

## 1 INTRODUCTION

In many low‐income countries (LICs), health systems remain weak and unable to cover the basic needs of their population or to cope with emerging threats or shocks such as outbreaks. The 2013‐2016 Ebola virus outbreak in Guinea, Liberia and Sierra Leone highlighted the fragility of the health systems in these countries and the Regions.^1^ Many studies have described the determinants of these weak health systems.^2^, ^3^, ^4^, ^5^ One of them is that they are organized as bureaucracies, not as learning systems.^6^ There is little attention to knowledge management and data intelligence. For instance, health information systems (HISs) management produces large amounts of data, mainly pulled by national programs for their own needs at the central level with little use at the decentralized level for health decision‐making.^7^, ^8^

There are alternatives. In Dakar in 2013, participants to a conference on the 25th anniversary of the Harare Declaration reconfirmed the validity of the health district strategy but also highlighted a need for a renewed vision.^9^ They proposed 12 key priority actions, among which the use of Information and Communication Technologies (ICT) to enhance governance and accountability, equity, effectiveness and efficiency of local health systems, and the promotion of constant learning at the district level to adapt strategies and intervention to their—specific, complex and constantly changing—environment.^10^

Even though electronic tools and eHealth initiatives have been highly used in recent years in resource‐limited settings,^11^ these initiatives mainly focused on improving the knowledge and behavior of the general population, the uptake of health care interventions by users,^12^, ^13^ the improvement of the relation between patients and health staff,^14^ the continuous education of clinical health professionals,^15^ or the data collection and transmission at the upper level.^16^ Rare are eHealth initiatives aiming at building “collective intelligence” at the district level. We miss documented experiences showing how to engage health district management teams (HDMTs) led by district medical officers (DMOs) into learning processes.

In this article, we present lessons from an action research project focused on the development and piloting of a new approach aiming at enhancing virtual exchange of knowledge—tacit and explicit—between HDMTs within a specific country. The approach is built on an online platform (we called it District.Team) and a specific knowledge process facilitation, as part of a broader eHealth initiative that we called Mobilization 2.0. We have piloted this approach in two countries: Benin and Guinea.

Our main hypothesis was that data collection, analysis, visualization and discussion between peers is feasible in resource‐limited settings and could help to develop learning and better performing local health systems. We hypothesized that a virtual environment where DMOs share data and experiences could be valued and help them to learn from each other and improve the performance of their local health systems.

The objectives of this article are to assess the participation of HDMTs in online knowledge exchange, identify enablers and barriers for mobilizing them using ICTs in technology constrained contexts and draw lessons for developing a learning local health system through digital platforms.

## 2 METHODS

### 2.1 Study design

Our investigation was organized along an action research design^17^ using a mixed methods approach (quantitative and qualitative). This research methodology was indicated, given the high level of uncertainty of the intervention. Indeed, the intervention was novel and complex and dependent on factors such as the environment, the attitude of stakeholders, the technology, the functionalities to integrate in the platform and the facilitation process.

We developed a conceptual framework for data collection and analysis. We adapted the framework designed by Aarts et al^18^ to analyze the barriers and facilitators of online health community tools. We added some of the criteria described by Barnett et al^19^ on joining a virtual community of practice. These criteria include the usefulness of the community, the clear definition of goals, the creation of a supportive environment, the benchmarking and the quality of the facilitation. Some criteria such as the context characteristics used by Murray et al^20^ to explain the difficulties in implementing eHealth initiatives, and of users' profiles of online platforms from Nijland et al^12^ were also added. Finally, some criteria on social networking and openness for participation proposed by Eysenbach et al^21^ were used.

### 2.2 Overview of District.Team

**FIGURE 1:**
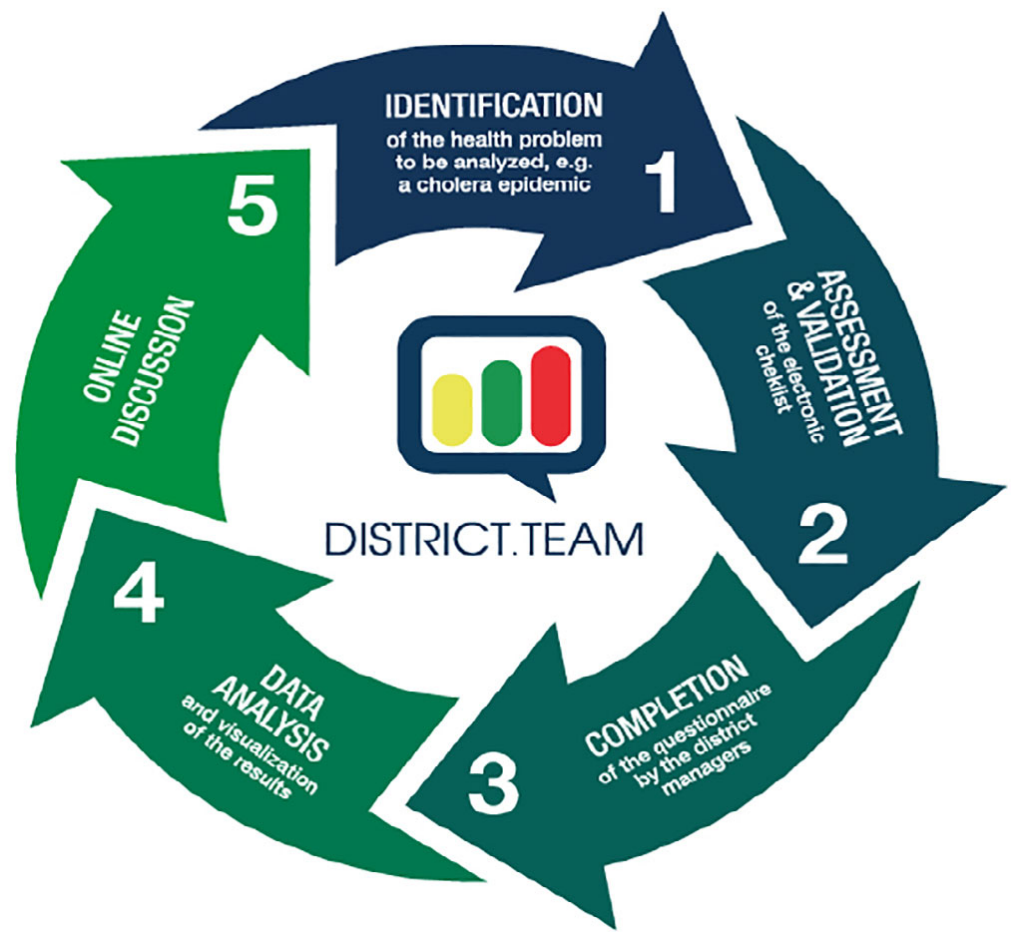
Steps of the collective learning process on District.Team

District.Team was a component of the Project “Mobilization 2.0 of HDMTs to fight against outbreaks and other emerging health issues,” funded by Unicef West and Central African Regional Office (WCARO) and implemented between January 2016 and September 2017 in Benin (a country not affected by Ebola) and Guinea (one of the most affected countries in West Africa). Benin and Guinea have 34 and 38 health districts, respectively. Each country had a project coordination team based in a research institution (Centre de Recherche en Reproduction Humaine et en Démographie in Benin and Centre National de Formation et de Recherche en Santé Rurale de Maferinyah in Guinea). Our main hypothesis was that there exists an interventional package partly relying on new technologies which can enhance real time exchange of knowledge between HDMTs for better district performance. Due to limited project time, we used a fast iterative process.^22^ District.Team as a collective learning process was developed by adapting criteria described by Blank and Dork^23^ for effective online platforms. It was organized in a cycle of five major steps (Figure 1):

1. Identification of a health issue to investigate: the health issue was purposely identified either by the research/facilitation team or by the DMOs (eg, the fifth cycle on maternal deaths surveillance and response), based on the principle of majority.
2. Elaboration of the online questionnaire by the facilitation team: the questionnaire aimed to document the practices on the field in relation with the national guidelines and to explore resources, activities and processes needed for an optimal response to a specific health issue by the local health systems. The online version of the questionnaire was developed using the Google form tool.
3. Administration of the questionnaire: The link of the online questionnaire was sent by email to DMOs for them to complete the questionnaire. Additionally, phone calls and SMS were used to interact with DMOs.
4. Data analysis, production and publication of results: Data were further analyzed and visualizations were produced by the facilitation team. The online assessment of the capacity of the local health systems to address the health challenge unveiled both their weaknesses and strengths. The visualizations were tables, graphs, maps or illustrations built using D3js (https://d3js.org/) and Carto (https://carto.com/) software. The visualizations were published online at country platforms (http://benin.district.team/ for Benin and http://guinee.district.team/ for Guinea). Each country has its own platform to facilitate in‐country interaction and exchange.
5. Online discussion forum on results: DMOs were invited to comment the results and to share their experience and thoughts. Discussions were guided by the facilitation team. Facilitators summarized the key lessons of the cycle that were also used to improve the following cycle and propose solutions to address the challenges.

During the Project implementation, in each country, five rounds were carried out by the project team. The first and second rounds focused on health district characteristics (such as the population size, the number of health areas, the availability of electricity and internet) and human resources, respectively. The third round started with online discussion on performance‐based financing in Benin and on obstetric fistulae in Guinea, for both we used results of recently published reports in Benin^24^ and studies in Guinea.^25^, ^26^ The fourth round was on epidemiologic surveillance in both countries. The fifth round analyzed the maternal death surveillance and response in both countries but started in Guinea with data collection through the online checklist and in Benin with the online discussion.

### 2.3 Study population and sampling

For the quantitative strand, we conducted an exhaustive sampling by including the 34 and 38 DMOs in Benin and Guinea, respectively. As for the qualitative strand, HDMT members including DMOS were purposely selected to participate to nine focus group discussions (FGDs) and 18 semi‐structured in‐depth interviews (IDIs).

### 2.4 Study variables

Quantitative variables included the visits on the platform, the participation of HDMTs for each cycle through the filling of the electronic questionnaires, commentaries on visualizations and experience sharing in the online discussion forum. They enabled to assess the participation of DMOs on knowledge exchange.

Qualitative variables were related to the facilitation, the enablers and disablers, the added value, the strengths and weaknesses of District.Team. This qualitative strand allowed to improve our understanding of barriers and enablers of HDMTs mobilization, the added value of District.Team and avenues for improvement.

### 2.5 Data collection procedures and period

Data were collected in each country through routine and active processes (Table 1).

**TABLE 1:** Conceptual framework of data collection

Criteria	Data	Source
*Facilitation*		
Processes of facilitation Incentives (internet) Mobilizing tools (email, phone call, SMS, electronic platform) Facilitation characteristics Profile and expertise of facilitators Knowledge sharing Overcoming professional isolation	Qualitative	Concept note Intermediate project's reports Semi‐structured IDIs, FGDs
*Context*		
Availability of the internet Electricity	Quantitative	Electronic database of Cycle 1
*Participation of HDMTs*		
Data collection (filling of the online questionnaire) Commentaries Online discussion forum	Quantitative	Intermediate project reports Electronic platforms (eg, Google analytics)
*Barriers and facilitators of HDMTs participation*		*National workshop*
Related to the intervention Related to participants Related to the facilitation	Qualitative	Intermediate project reports Semi‐structured IDIs, FGDs
*Added value of District.Team*		*National workshop*
Human resources Health information system management	Qualitative	Semi‐structured IDIs, FGDs
*Strengths and weaknesses of District.Team*		*National workshop*
Strengths Weaknesses Lessons learnt	Qualitative	Semi‐structured IDIs, FGDs

Abbreviations: FGDs, focus group discussions; HDMTs, health district management teams; IDIs, in‐depth interviews; SMS, short message service.

Firstly, routine data were mainly quantitative and were collected on the District.Team platforms (WordPress and Google Analytics statistics) during the implementation of the project.

Secondly, the active data collection was mainly qualitative and was done at the end of the project in June 2017. We conducted nine FGDs and 18 semi‐structured IDIs with DMOs during five and four regional workshops organized in Benin and Guinea, respectively. All DMOs and one to two HDMT members were invited to the workshop. The FGDs and IDIs were carried out by the District.Team coordinator (Kéfilath Olatoyossi Akankè Bello) and a socio‐anthropologist in Benin and by the two project coordinators in Guinea (Tamba Mina Millimouno and Sidikiba Sidibé).

### 2.6 Analyses

Quantitative data were extracted from WordPress and Google Analytics and analyzed using the Excel 2016 software. Descriptive statistics were summarized as proportions. The FGDs and IDIs were fully transcribed into French and analyzed using a thematic coding based on the conceptual framework.

### 2.7 Ethical considerations

We obtained Ethical clearance from the National ethics committees of Benin and Guinea. The Ministry of Health of Benin issued a written administrative authorization to conduct the project. In Guinea, we received a verbal consent to conduct the project from the Ministry of Health. The Institutional Review Board of the Institute of Tropical Medicine, Antwerp approved the Project. The objectives of the project and the research were explained to DMOs prior to its start. All participants to the focus groups and interviews signed an informed consent. Data related to perception and views of participants were analyzed and kept confidential.

## 3 RESULTS

In this section, we present the key results obtained during the process of the action research during the project with some data published on the two websites (http://benin.district.team/ for Benin and http://guinee.district.team/ for Guinea) and the perception of key participants collected at the end of the project.

### 3.1 Context

**FIGURE 2:**
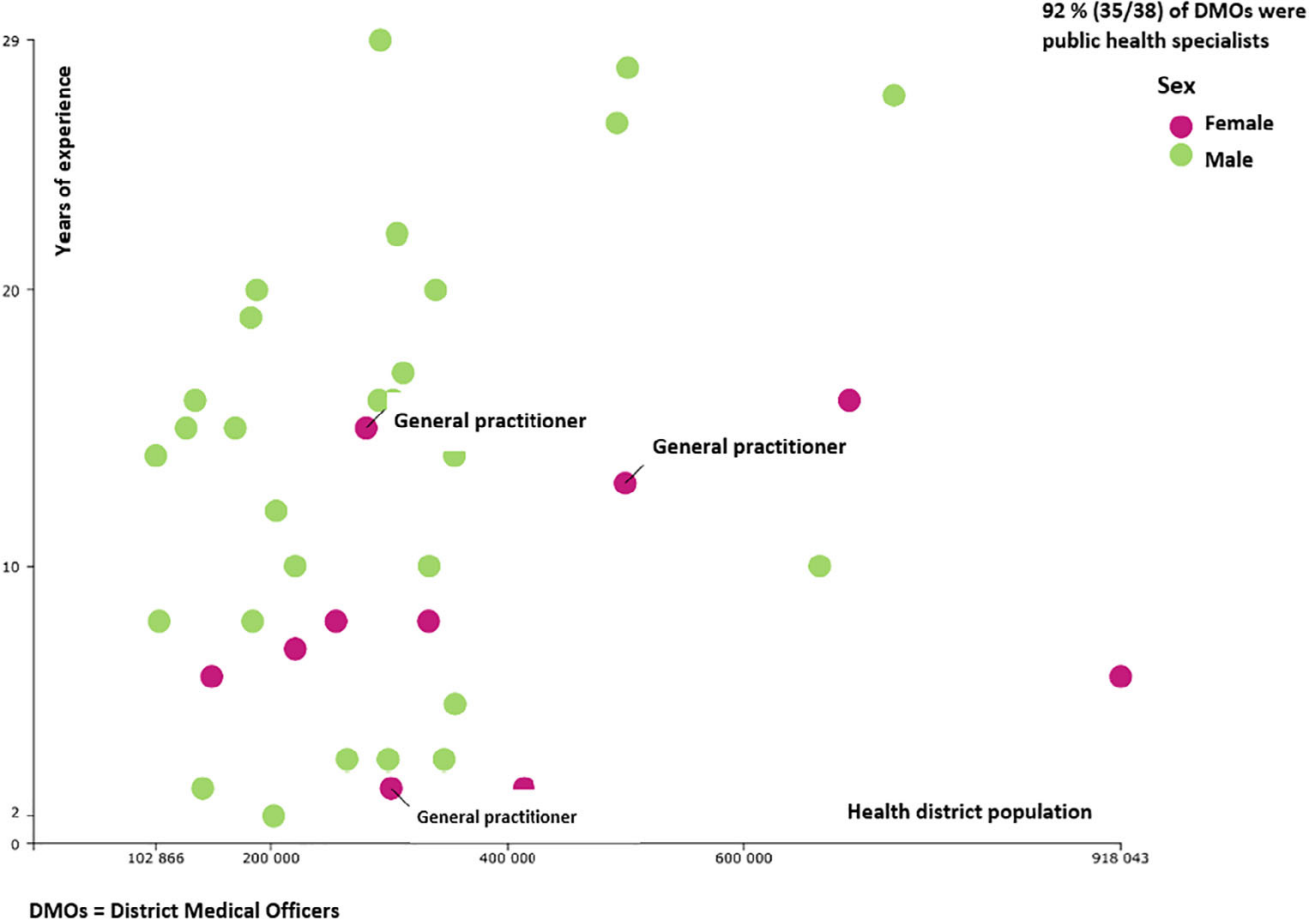
Profile of district medical officers and their districts in Guinea in 2016. The illustration shows the profile of district medical officers (specialization, sex and years of experience) correlated to the health district population in 2016

During the cycle 1, we collected some information on connectivity and profile of DMOs in both countries. In Guinea, only 36% of health districts had full mobile phone coverage and an acceptable to good internet connection. Most (58%) of the health districts had partial mobile phone coverage. In Benin, 57% of health districts had full mobile phone coverage and an acceptable to good internet connection. In the two countries, more than 90% of health districts were run by a medical doctor with a Master in Public Health (Figure 2).

**FIGURE 3:**
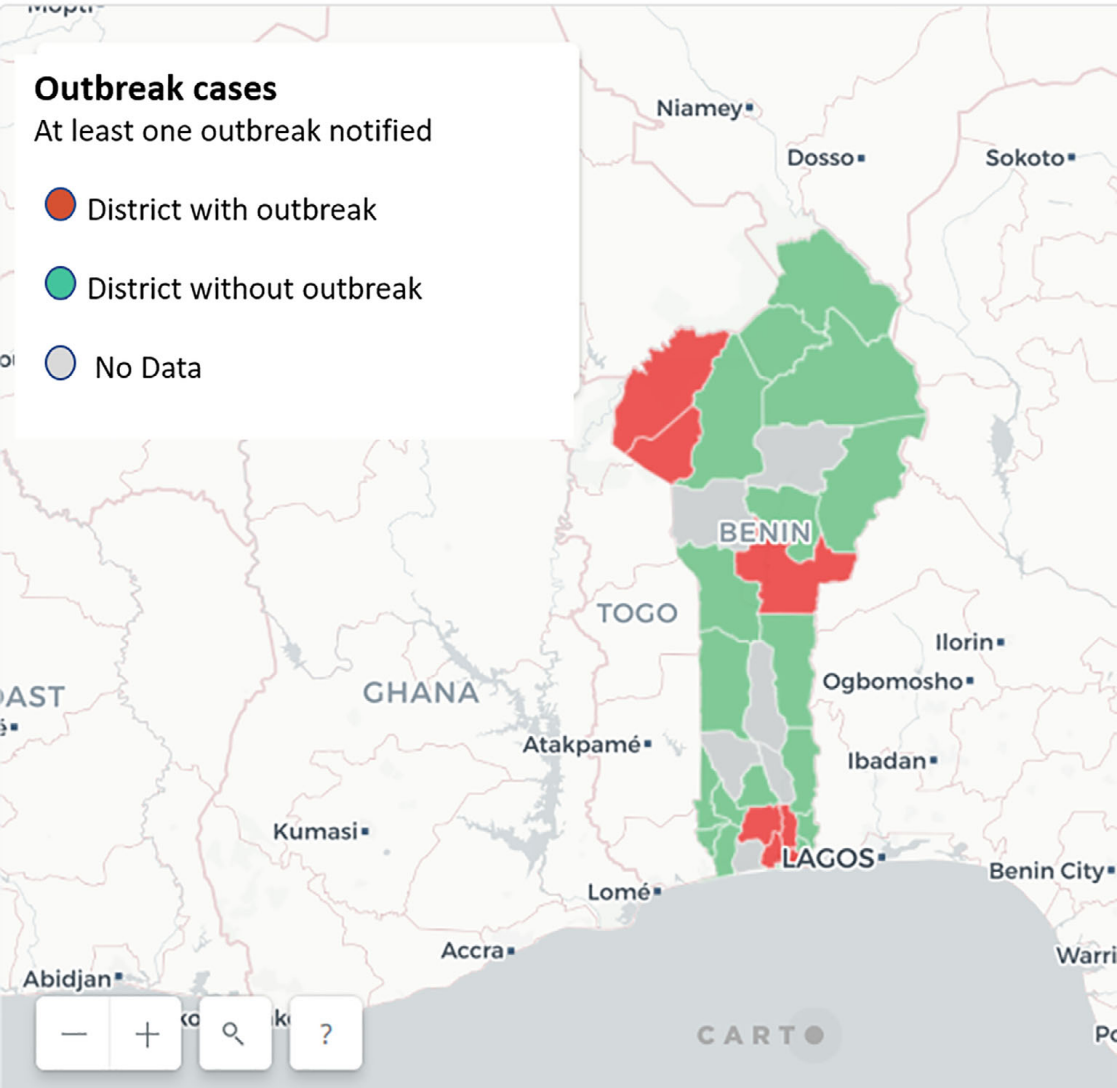
Outbreak situation in Benin health districts in 2016. Benin's map showing health districts that notified a case of a disease under surveillance in 2016

In 2016, 76% (29/38) of health districts registered outbreaks in Guinea while only 15% (5/34) of districts notified an outbreak in Benin (Figure 3).

### 3.2 DMOs' mobilization

Participation of DMOs in filling the online checklists varied from 79% to 84% for the rounds 1, 2 and 4 in Benin, and from 61% to 100% for the rounds 1, 2, 4 and 5 in Guinea.

In Benin, 14 (41%) and 9 (26%) DMOs commented on the platform the results of cycles 1 and 2, respectively while 7 (21%) DMOs shared their views using the online discussion forum for the two cycles. In Guinea, the comments were made only by 3 (8%) and one (3%) DMOs for the cycles 1 and 2, respectively while 15 (40%) and 4 (11%) DMOs shared their points of views using the online discussion forum for the cycles 1 and 2, respectively. For cycle 3, 13 (38%) and 14 (37%) DMOs participated in the online discussion forum in Benin and Guinea, respectively (Table 2).

**TABLE 2:** Mobilization of HDMTs per country

Round	Health issue	Length of the cycle (days)	HDMTs who participated in		
			Filling the checklist	Commenting the results	Online discussion
Benin					
1	Health district characteristics	132	29 (85%)	14 (41%)	7 (21%)
2	Human resources	92	32 (94%)	9 (26%)	7 (21%)
3	Performance based financing	51	—	—	13 (38%)
4	Epidemiologic surveillance	135	27 (79%)	—	16 (47%)
5	Maternal deaths surveillance and response	44	—	—	15 (44%)
Guinea					
1	Health district characteristics	125	38 (100%)	3 (8%)	15 (40%)
2	Human resources	58	37 (97%)	1 (3%)	4 (11%)
3	Obstetric fistulae	125	—	—	14 (37%)
4	Epidemiologic surveillance	126	27 (79%)	—	3 (8%)
5	Maternal deaths surveillance and response^a^	123	23 (61%)	—	28 (74%)

Abbreviation: HDMTs, health district management teams.

^a^ For Guinea, beyond the District.Team platform, the results of this cycle have been also published elsewhere.^27^

### 3.3 Use of District.Team platforms (users, sessions, page views…)

Between July 2016 and July 2017, 777 and 608 users visited the Benin and Guinea District.Team platforms, respectively. Though, there were more sessions and pages views in Benin than in Guinea. On the contrary, Guinean users spent more time per session (Table 3).

**TABLE 3:** Use of the District.Team platform between July 2016 and July 2017

Indicators	Benin	Guinea	Mean per district	
			Benin	Guinea
Number of health districts	34	38	—	—
Users	777	608	23	16
Number of sessions	2703	1602	80	42
Number of pages seen	11 918	8902	351	234
Rebound rate	17.17%	10.47%		
New visitors	28.23%	35.71%		
Number of pages seen per session	4.41	5.40		
Mean length of a session	5mn	6mn 12 seconds		

### 3.4 Enablers and barriers for mobilizing DMOs through District.Team

Enablers and barriers for mobilizing DMOs through District.Team were related to the intervention, participants and the facilitation.

### 3.5 Enablers related to the intervention

The virtual asynchronous nature of District.Team with limited face‐to‐face activities was noted by DMOs as the main strength, as each member could participate at any time and place. Moreover, this participation was freely accessible through internet. One participant declared that “*there are less face‐to‐face meetings, you do not need to travel to participate*” (Benin, Focus Group). They stressed that District.Team was innovative with a user‐friendly platform where all data and members' contributions are shared. Allocation of internet connection fees facilitated connection and exchange even beyond District.Team as declared by one interviewee: “*Internet connection fees also helped us to send our reports to the hierarchy and respond to our mails*.” The quality of the mobile phone network coverage also influences the participation. The nature of the theme analyzed was noted as an important factor; therefore, the theme should be relevant or address health district related issues. In Guinea, one DMO noted that “*the theme on the management of human resources was very interesting, essential and relevant as only 4 over 30 positions are filled by the government. We were keen to know what was the situation in other districts*.”

### 3.6 Enablers related to participants

The main factor cited by all interviewees and focus group participants was the perception of the usefulness of District.Team, the familiarity with internet and computer, and the availability of a smartphone. For instance, many DMOs were interested by District.Team because it helps to share their own experience and to learn from peers.

### 3.7 Enablers related to the facilitation

During the project, we found that using multiple mobilization tools such as emails, phone calls or SMS to invite and send reminders to the participants was a best practice to enhance the participation to the project. Moreover, it was also important to draw and share the lessons learnt at the end of the cycle. One DMO in Guinea insisted on the support for filling the checklist and for navigating the platform especially at the start of the project. Additionally, it was highlighted during focus groups that the availability and the courtesy of facilitators, and the use of experts to respond to DMOs questions and concerns facilitated understanding and exchanges. Finally, it was stressed that face‐to‐face meetings were necessary at the start of the project to clearly discuss the objectives, activities and expectations of all stakeholders.

### 3.8 Barriers related to the intervention

As the online discussion forum was not open‐access, some DMOs had difficulty in creating their profile and regularly complained that they had lost their password. All DMOs reported the electricity irregular supply and the instability and low quality of the internet connection as the main barriers to the intervention. In Guinea, one DMO declared that “*the quality of the internet connection limited my participation. Regarding electricity, I do not even have it; I use an electric generator that needs 20 liters of fuel per day, but I do not have any subsidy*.” Another DMO from Guinea added that “*sometimes, you have an electronic failure, your office computer shuts down and you lose what you were doing*.”

Participants of the focus group insisted that there was an inadequate link between the project and the central level. They noticed that they have lot of pressure to undertake and fulfill the requirements from the central level such as transmission of reports or participation in seminars. Consequently, the centrally mandated activities are prioritized at the expense of district's priorities.

### 3.9 Barriers related to participants

During the project, we noted that the participation was associated with younger age of the DMOs—this seems to relate to digital literacy. All DMOs revealed that barriers related to participation also include the lack of time and interference with other solicitations by the regional and central staff, vertical programs, financial and technical partners. One DMO from Guinea noted that “*with lot of administrative constraints, you do not even have time to read your emails on time*.” Another DMO from Benin added that “*we use our free time to participate to District.Team*.” It appeared during FGD that some DMOs are not familiar with the use of computer and internet. Many DMOs acknowledged that the low participation of the central level did not give them confidence to share their views in a public forum. A DMO from Benin said that “*what was lacking was the participation of the central level; if my hierarchy is not interested in this project, why should I be?*” Lastly, some DMOs reported that they were already registered in many online professional fora; some forgot their District.Team username and password so they could not participate anymore.

### 3.10 Barriers related to the facilitation

A major weakness of the facilitation identified by interviewees and focus group participants was the irregular synthesis of the lessons learnt during some cycles. Indeed, a blog was published by the research team only for the three last cycles to describe the main outcomes and how HDMTs can improve their performance on the specific issue. Other factors were: the irregular allocation of internet fees, and the insufficient communication on District.Team's objectives, procedures and key steps. DMOs also pinpointed that the facilitation team did not propose concrete solutions to the problems affecting health districts' performance that were identified during cycles (Table 4).

**TABLE 4:** Enablers and barriers of DMOs' mobilization through District.Team

Level	Enablers	Barriers
Related to the intervention	Free and simple online platform; individual funding for internet fees; online archiving of exchanges for future access; data requested already available via the routine health information system; virtual design of the project; issues analyzed were relevant to the district level	Irregular electricity supply; poor internet connection; poor design of the electronic checklist; little implication of the central level Poor communication of the objectives, procedures and content of the project No implication of DMOs in the design of the Initiative and selection of the issue to be analyzed Few solutions to address the identified problems; too long delay between some cycles; the platform has too many links to access information Short duration of the project; some visualizations were complex and difficult to understand
Related to participants	Perception of the usefulness of District.Team; knowledge of internet and computer; perception of District.Team as a learning and experience sharing tool; participation of other colleagues; willingness to share personal experience	Lack of time; interferences with other activities Username and password forgotten Fear to publicly share personal views online; multiplicity of online fora; limited involvement of other members of the HDMTs by DMOs; little attention to activities not followed by the hierarchy
Related to the facilitation	Frequent reminders (SMS, emails, WhatsApp, phone calls); use of opportunities of some face‐to‐face events to present District.Team; diffusion of other useful information for DMOs	Irregular synthesis of the lessons learnt per cycle Limited face‐to‐face meeting to explain the District.Team concept and design; irregular and poor communication with districts; communication limited to DMOs Irregular and insufficient funding for internet connection fees; thematic chosen by the facilitators without implication of DMOs Some blogs were too long; planning of the cycle not shared with DMOs

Abbreviations: DMOs, district medical officers; HDMTs, health district management teams; SMS, short message service.

### 3.11 Effects of District.Team

District.Team is perceived as a tool for exchanging knowledge and sharing experiences as it improved interactions between peers. A DMO noted that “*with District.Team, we became aware that each DMO has developed specific skills and competencies and we could learn from each other*.” It also contributed to improving health district performance. For examples, a DMO from Benin said that “*Thanks to data visualization, we identify weaknesses in our districts and try to address those that are under our responsibility*”; or “*in my district, due to the cycle on disease surveillance, we analyzed our situation and improved our preparedness to cholera outbreak* (DMO from Guinea).” District.Team served as a benchmarking tool as highlighted by a DMO from Benin “*District.Team helps to compare health districts, and you can discover that some districts perform more poorly than yours*.” Finally, the initiative contributed to learning as noted during focus group in Benin “*experts' contribution helped to correct misunderstanding that I have on performance‐based financing*.”

## 4 DISCUSSION

With this action research, we have confirmed that it is possible to enhance, through digital means, knowledge exchange among HDMTs in LICs such as Benin and Guinea. To our knowledge, there was no other similar experience in resource‐limited settings to build upon; the action research methodology helped us to iteratively improve our approach and digital solution. We have confirmed that DMOs are ready to participate to some collective health system analysis based on primary or secondary data and produce “collective intelligence” in a horizontal manner.^28^ We believe that with this experience, a “proof of concept” is emerging. It encompasses adaptation and learning as this approach is influenced by barriers and enablers highlighted by other studies on health‐related online communities.^18^ Real time exchange and sharing of experiences by HDMTs are feasible in resource‐limited settings using online platforms through online data collection, analysis and publication of results, commentaries on results, and online discussion forum.

We have learnt several key lessons. District.Team is the exploitation of two types of knowledge—data (explicit knowledge) and the experience and expertise of people (tacit knowledge). Four key elements emerged from our experience: the digital tools, the facilitation, participants, and the content and data management. We believe that these four elements are key components required for collective intelligence. Our analysis revealed the importance of a fifth one that we missed during the implementation phase: leadership from legitimate authorities.

### 4.1 The digital tools

Our experiment confirmed that there is room for some digitalization of the interaction between DMOs. In vast countries with poor road infrastructure, we must move beyond the practice of face‐to‐face meetings. Even though improving the competencies of health staff is important, many experts highlighted that in many LICs, workshops and seminars weigh on the performance at the decentralized level, as they pull health staff from their workplace.^29^ Our recommendation is not to drop all face‐to‐face meetings—they are key to create trust and collegiality, but to combine them with solutions like District.Team, which can effectively connect DMOs in‐between meetings and workshops. The tools we used are quite generic and low‐cost: Google forms for data collection, a dedicated website for data publication, online discussion forum, emails, mobile phone short messages and phone calls.

The District.Team website, the open‐access platform where results of a cycle, commentaries and discussions are published, offers a room for more transparency and accountability in the management of local health systems. In addition, it could become part of the solution to address the bottlenecks of bureaucracy and high centralization that characterize some weakest sub‐Saharan African health systems.

However, the opened‐platform of District.Team could also be seen by some managers—especially those with poor results—as a threat. In Tanzania, putting in place mechanisms of transparency and accountability was seen by some HDMTs' members as a threat.^30^ An option for the future could be to integrate some of the features of District.Team into existing routine HIS platforms such as DHIS2. Indeed, data pulled by DHIS2 could be analyzed automatically or by the central level staff and results published on the platform in terms of graphs, maps and tables comparing health districts. Then, the facilitating team of each program would propose subjects for online discussion to identify practical solutions for improving health districts' performance.

### 4.2 Facilitation

One cannot stress enough the importance to establishing centralized capacity to facilitate the online interactions. We have tested a model relying on researchers based in national research institutes. Peer‐to‐peer experiential learning rarely emerges spontaneously. There are techniques and tools to enhance exchanges. We have used a cyclic approach. The results of the cycle were used to inform and possibly improve the next cycle. Monitoring DMOs' participation was a core task of the facilitation team. The facilitation team should have enough time to interact with DMOs and improve their interest and motivation. Even though e‐solutions appear to be cheaper for the beneficiaries, it needs a dedicated and skilled team that masters both the health issues under discussion and facilitation techniques.^31^

### 4.3 Participation of DMOs

Collective intelligence in health districts requires adhesion from DMOs. Their participation is influenced by their interest, motivation and the perceived relation between the project activities and their daily work. We have learned several lessons in this respect. The model should for instance avoid increasing the workload of the people involved and align its activities and outputs as much as possible to their routine work. DMOs should be exclusively in charge of the selection of issues for greater ownership of the process. The online questionnaire could be used to primarily identify the key issues facing health districts.

### 4.4 Content and data management

Our group had previous experience with facilitation of online discussion fora—our assessment is that conversation only does not suffice for the development of sound collective intelligence. Collective intelligence is stronger when it rests on data (vs tacit knowledge only). District.Team relies on analyzing district data, then publishing visualizations on the results achieved by HDMTs as a key strategy for benchmarking. It contributed to data analysis and sharing, discussion and exchange, as first steps for decision‐making. We made a clear choice for a co‐production approach to data and empowerment through data. This can be opposed to most HISs' initiatives in low‐income countries which are designed to *pull* health data from communities or health facilities to the district, regional and central levels of the health system.^32^, ^33^, ^34^ In this context, data are rarely analyzed and used for decision‐making at the operational level, and are not accessible to peers.^23^ On the contrary, District.Team tries to help local health systems to become learning organizations. Our vision is that such systemic learning capacities are key for building strong health systems.^35^, ^36^, ^37^ Even though the whole health system is weak, there are HDMTs that perform better. However, their results are not always publicly known, as they are lost in the aggregated national data. By enabling staff to focus on particularities at the district level, a solution like District.Team offers an avenue to explore complexity of health systems. It provides a room for peers to know “who does what,” “what are the challenges and solutions used to address them,” “what results were achieved in each district” and “How did peers achieve results.”

### 4.5 Leadership

We have identified one weakness in our approach: it was not supported enough by the national health authorities. In highly centralized health systems such as in Benin and Guinea, HDMTs rely mostly on directives from the central level.^38^ The institutional endorsement and integration of District.Team into existing programs were not strong enough. Indeed, District.Team was initially designed as a parallel initiative to the routine HIS; it was managed by a research institution and inevitably contributed to increase the workload of already burdened DMOs. Some constraints that we faced could probably be avoided if the initiative was institutionalized and if senior officials of the MoH followed or even contributed to the online exchange. Collective learning approaches should ideally be integrated in the routine management of the health system, at all its different levels.

Asamoah‐Odei et al^39^ proposed to strengthen leadership and coordination as well as the capacity of the health sector to lead the process of eHealth initiatives. The literature on “learning organization” also stresses the pivotal role of leadership: decision‐makers should send a strong signal that learning (with its part of critical review, unorthodox thinking…) and adaptation are key to health system performance and develop a conducive environment for learning.^37^ The central level can better put in place and sustain at large scale the 3Ms—meaning, management and measurement—proposed by Garvin for building a learning organization.^40^

### 4.6 Limits

We faced several constraints that limited the optimal implementation of District.Team, such as poor internet connection and the irregular supply of electricity. Most of these factors are also determinants of weak health systems^4^; they should be considered and tackled when introducing District.Team in a country.

District.Team has also several limits. Firstly, the Initiative lasted for only 14 months so it was not possible to capture its long‐term effects. The two research institutions are seeking for resources to sustain District.Team for exploring other health issues. Secondly, we have only two study countries, therefore, some results are specific to these countries. Thirdly, a baseline study of the mobilization of DMOs was not carried out before the intervention and effects described may not be solely linked to District.Team. Fourthly, District.Team was a funded and facilitated research project and such a support may not be available in limited resource settings. It was also implemented as a parallel initiative and focused primarily on mobilizing DMOs rather than solving problems identified during each cycle as many solutions proposed by DMOs required the central level interventions.

However, despite these limitations, the study provided useful insights for digital mobilization of DMOs without over‐increasing their burden in resource‐limited settings. Even though each sub‐Saharan African country has its own specificities, many countries are still characterized by their weaknesses and inability to address population needs. Therefore, initiatives aiming at strengthening health systems from some countries can be adapted and implement elsewhere. We suggest that lessons learnt from District.Team could inform digital initiatives elsewhere and encourage others to explore the avenue for platforms dedicated to sharing knowledge and best practices among HDMTs. This could contribute to stronger local health systems better equipped to address populations' needs.^41^ Utmost, to integrate at scale eHealth initiatives in health care organizations, organizational capabilities are needed across three domains: policies and processes (content and data management), technology (digital tools), and people (participation of DMOs, facilitation, leadership).^42^ Overall, granting a much more central role to “learning” in health system strengthening efforts is crucial^43^ and transforming health systems into learning organizations need to be given high priority in countries' efforts to achieve universal health coverage as well as the sustainable development goals.^43^, ^44^ The District.Team initiative nicely fits in this new orientation and is an example of possible practical steps.

## 5 CONCLUSION

District.Team has shown some potential for improving knowledge management, developing learning organizations and building collective intelligence. Therefore, lessons learnt from this study could be applied not only in resource‐limited settings but in all health systems to address the complexity and adapt interventions and strategies to the changing environment. There is room for peer‐to‐peer knowledge sharing across HDMTs and more *bottom up* approaches for addressing health system issues. Factors such as electricity, ICT equipment and internet connection are still constraints in some health districts, but one can expect they will be removed with the ongoing infrastructure development. To institutionalize such initiatives, it is probably more important to focus on investing in the facilitation capacity and on securing support from the national health authorities. From a technological perspective, different avenues are possible, including integrating digital features and facilitation capacities into existing HIS platforms.

## CONFLICT OF INTEREST

Bruno Meessen holds minority shares in Blue Square. Pierre Massat owns Mavromatika.com. Other authors declare no conflicts of interest.

## AUTHOR CONTRIBUTIONS

All authors contributed to the protocol design, data collection and analysis and to the revision of the manuscript up to the final version. Basile Keugoung wrote the first draft of the manuscript.
